# Development of an Anti-canine PD-L1 Antibody and Caninized PD-L1 Mouse Model as Translational Research Tools for the Study of Immunotherapy in Humans

**DOI:** 10.1158/2767-9764.CRC-22-0468

**Published:** 2023-05-15

**Authors:** Wonkyung Oh, Alyssa Min Jung Kim, Deepika Dhawan, Perry M. Kirkham, Raluca Ostafe, Jackeline Franco, Uma K. Aryal, Robert H. Carnahan, Valery Patsekin, J. Paul Robinson, Deborah W. Knapp, Seung-Oe Lim

**Affiliations:** 1 Department of Medicinal Chemistry and Molecular Pharmacology, Purdue University, West Lafayette, Indiana.; 2 Department of Veterinary Clinical Science, Purdue University, West Lafayette, Indiana.; 3 Office of the Executive Vice President for Research and Partnerships, Purdue University, West Lafayette, Indiana.; 4 Molecular Evolution, Protein Engineering and Production, Purdue Institute for Inflammation Immunology and Infection Diseases, Purdue University, West Lafayette, Indiana.; 5 Purdue Proteomics Facility, Bindley Bioscience Center, Purdue University, West Lafayette, Indiana.; 6 Department of Comparative Pathobiology, Purdue University, West Lafayette, Indiana.; 7 Vanderbilt Vaccine Center, Vanderbilt University Medical Center, Nashville, Tennessee.; 8 Department of Pediatrics, Vanderbilt University Medical Center, Nashville, Tennessee.; 9 Department of Basic Medical Sciences, Purdue University, West Lafayette, Indiana.; 10 Weldon School of Biomedical Engineering, Purdue University, West Lafayette, Indiana.; 11 Purdue Institute for Cancer Research, Purdue University, West Lafayette, Indiana.; 12 Purdue Institute for Drug Discovery, Purdue University, West Lafayette, Indiana.

## Abstract

Immune checkpoint blockade therapy, one of the most promising cancer immunotherapies, has shown remarkable clinical impact in multiple cancer types. Despite the recent success of immune checkpoint blockade therapy, however, the response rates in patients with cancer are limited (∼20%–40%). To improve the success of immune checkpoint blockade therapy, relevant preclinical animal models are essential for the development and testing of multiple combination approaches and strategies. Companion dogs naturally develop several types of cancer that in many respects resemble clinical cancer in human patients. Therefore, the canine studies of immuno-oncology drugs can generate knowledge that informs and prioritizes new immuno-oncology therapy in humans. The challenge has been, however, that immunotherapeutic antibodies targeting canine immune checkpoint molecules such as canine PD-L1 (cPD-L1) have not been commercially available. Here, we developed a new cPD-L1 antibody as an immuno-oncology drug and characterized its functional and biological properties in multiple assays. We also evaluated the therapeutic efficacy of cPD-L1 antibodies in our unique caninized PD-L1 mice. Together, these *in vitro* and *in vivo* data, which include an initial safety profile in laboratory dogs, support development of this cPD-L1 antibody as an immune checkpoint inhibitor for studies in dogs with naturally occurring cancer for translational research. Our new therapeutic antibody and caninized PD-L1 mouse model will be essential translational research tools in raising the success rate of immunotherapy in both dogs and humans.

**Significance:**

Our cPD-L1 antibody and unique caninized mouse model will be critical research tools to improve the efficacy of immune checkpoint blockade therapy in both dogs and humans. Furthermore, these tools will open new perspectives for immunotherapy applications in cancer as well as other autoimmune diseases that could benefit a diverse and broader patient population.

## Introduction

Immune checkpoint blockade therapy, one of the most promising forms of cancer immunotherapy, has been successful in multiple cancer types, including invasive urinary bladder cancer, the focus of this study (1, 2). In particular, programmed cell death protein 1 (PD-1)/programmed death-ligand 1 (PD-L1) pathway blockade using anti-PD-1 or anti-PD-L1 antibodies has elicited durable clinical responses in patients with cancer, presumably by normalizing imbalances in antitumor immunity (3). Given the promising and durable clinical responses, the FDA approved three PD-1 antibodies, nivolumab, pembrolizumab, and cemiplimab, and three PD-L1 antibodies, atezolizumab, avelumab, and durvalumab, for multiple types of cancer in humans (4, 5). Although this worthy milestone conveys the excitement and promise of this novel form of cancer treatment, PD-1/PD-L1 blockade therapy in cancer is currently not satisfactory due to the limited response rates (20%–40%; refs. 3–5). Therefore, new immunotherapeutic strategies to improve the efficacy of current PD-1/PD-L1 blockade therapies are urgently needed.

Strategies to improve PD-1/PD-L1 blockade therapies in bladder cancer and other cancers include: (i) identifying host factors including genetics, immune state, and molecular subtype that drive a relevant response, (ii) assessing biomarkers and combinations of biomarkers to predict response and to personalize therapy, (iii) developing better tools to monitor immune effects, and (iv) selecting combination drug approaches/regimens to address multiple “defects” in the immune response in addition to PD-1/PD-L1 blockade. Relevant preclinical animal models are essential to developing these strategies and testing multiple combination approaches. Factors that are likely to affect the PD-1/PD-L1 axis and thus must be represented in animal models, include aggressive and metastatic cancer behavior, tumor heterogeneity, mutational landscape, genetic and epigenetic cross-talk, cancer molecular subtypes, immune cell responsiveness, and innate and acquired mechanisms of drug resistance. Experimental rodent models, including carcinogen-induced, engraftment, and genetically engineered models, are instrumental in research of different types of cancer including bladder cancer (6–9). However, rodent models lack the collective features that are critical to studying emerging therapies within and across molecular subtypes in bladder cancer, and to predicting therapeutic success or failure in humans.

While these collective features are lacking in rodent models, we have demonstrated that pet dogs with naturally occurring invasive urothelial carcinoma (InvUC; comprising >90% of bladder cancer in dogs) can provide this crucially needed relevant model in an immunocompetent host. The canine model can complement other models to drive preclinical research to understand and optimize drug activity in humans. Canine InvUC mimics human InvUC in presentation, pathology, local invasion, distant metastases (lung and other organs in >50% of cases), and chemotherapy response (10–20). Canine and human InvUC are similar in terms of druggable mutations, pathway variants, epigenetic targets, and transcriptomic patterns of molecular subtypes (basal, luminal; refs. 11, 13, 18, 21–27). InvUC represents 1.5%–2% of the estimated 4 million new cases of canine cancer annually in the United States, so ample numbers of dogs are available for translational studies (28). Canine clinical trials in which dogs continue life as pets are a win-win situation with benefits to each dog and knowledge gained to help people and pet dogs facing cancer (11, 14, 18). Thus, dogs offer an excellent opportunity to advance PD-1/PD-L1 blockade therapies in humans. Successful treatment approaches in rodents can be evaluated in dogs, and those that have the highest success can be moved into human trials.

Canine PD-1/PD-L1 blockade antibodies are not commercially available for dogs with InvUC. The development of canine PD-L1 (cPD-L1) antibodies has been described by several academic groups (29–33). Tumor regression in dogs with oral melanoma and soft-tissue sarcomas was reported in response to a canine chimeric mAb targeting PD-L1 (30, 33). The antitumor effects of this antibody, however, require further study as the effect of concurrent medications (30, 34, 35) on the tumor regression was not determined. In other work, Choi and colleagues developed anti-canine PD-1 and PD-L1 antibodies for diagnostic applications, but these have not been used therapeutically (31). Therefore, development of a canine immune checkpoint blockade antibody, that is, an anti-cPD-L1 antibody, for dogs is important for translational research.

In this study, we developed a new immunotherapeutic cPD-L1 antibody and characterized its functional/biological properties *in vitro* and *in vivo*. Furthermore, we validated the therapeutic efficacy of cPD-L1 antibodies using our newly established caninized PD-L1 mice, which express cPD-L1 on the cell surface. Our cPD-L1 antibody, a new immuno-oncology drug, and caninized PD-L1 mice will be essential translational research tools in raising the success rate of immunotherapy in dogs and humans.

## Materials and Methods

### Study Overview

**FIGURE 1:**
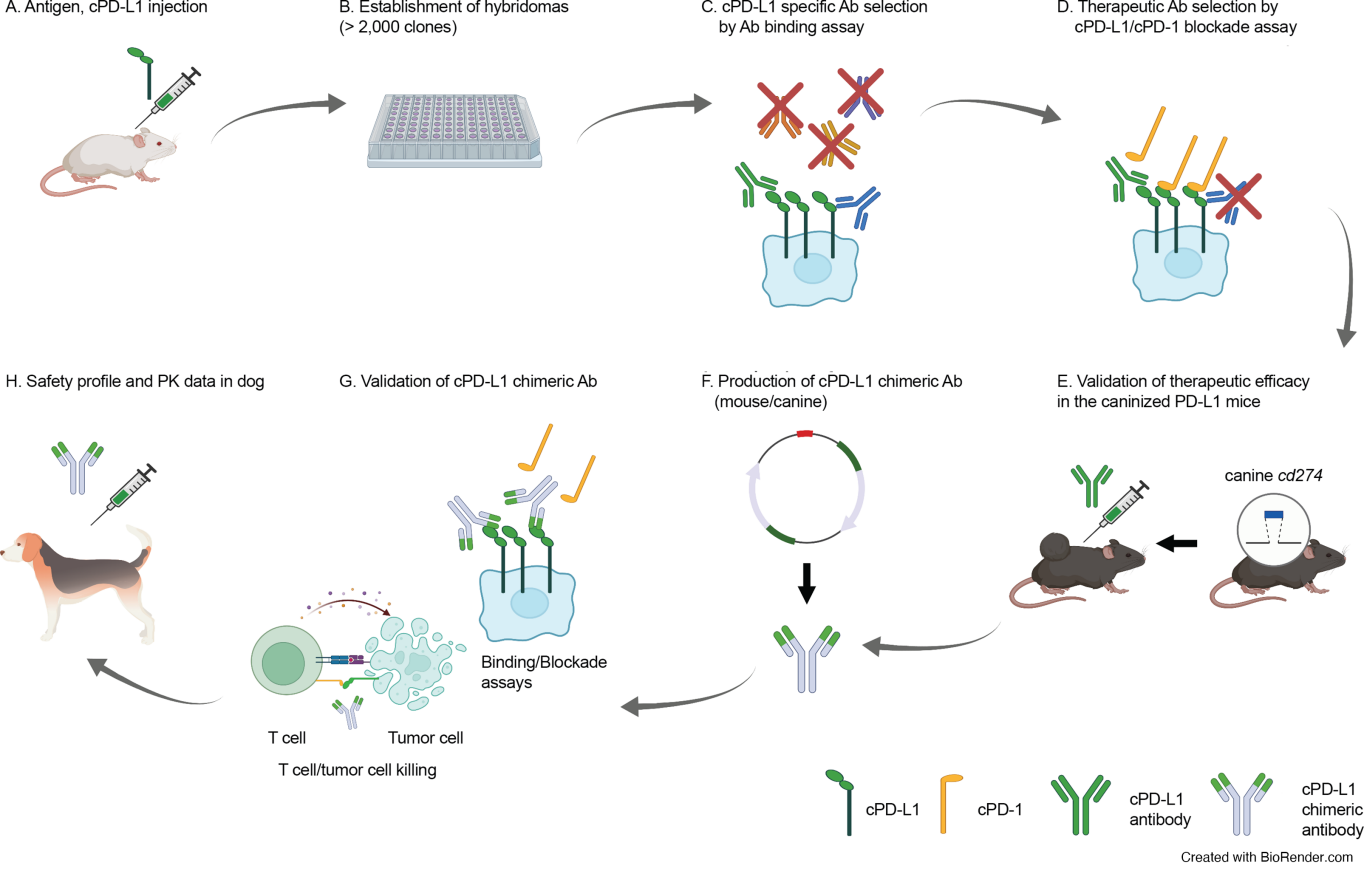
Working flow chart for production and validation of the cPD-L1 antibody. **A,** Immunization of antigen, cPD-L1 protein. **B,** Establishment of hybridomas (over 2,000 clones). **C,** Evaluation of the cPD-L1 specific antibody by the live cell–based antibody binding assay. **D,** Therapeutic antibody selection by cPD-L1/cPD-1 blockade assay. **E,***In vivo* validation of therapeutic efficacy of cPD-L1 antibodies in the caninized PD-L1 mice. **F,** Production of the cPD-L1 chimeric antibody. **G,** Validation of the cPD-L1 chimeric antibody. **H,** Initial safety profile and PK analysis. PK, pharmacokinetic.

The work included: (i) generation of cPD-L1 antibodies in mice, (ii) selection of the superior clone to further develop, (iii) evaluation of the cPD-L1 antibody in mice expressing cPD-L1 on the cell surface and implanted with tumor cells expressing canine PD-1, (iv) creation of a chimera antibody of the top clone, (v) characterization of the chimera antibody through cell-based and cell-free cPD-L1 antibody binding and cPD-L1/cPD-1 blockade assays, and other assays of essential characteristics, (vi) assessment of the antibody activity in an *ex vivo* canine peripheral blood mononuclear cell (PBMC)-mediated killing assay, and (vii) initial safety and pharmacology study in dogs, and is summarized in Fig. 1. All work involving animals was performed with approval of the Purdue Animal Care Use Committee.

### Cell Culture, Stable Transfectants, and Transfection

The BT549 human breast cancer and MB49 mouse bladder cancer cell lines were obtained from ATCC and Millipore Sigma, respectively. The canine bladder cancer cell line, K9TCC was obtained from the Dr. Deborah W. Knapp Lab (36). The human embryonic kidney cell line HEK293FT was obtained from Thermo Fisher Scientific. Cell lines were validated by short tandem repeat DNA fingerprinting using the AmpFlSTR Identifiler PCR Amplification Kit (Thermo Fisher Scientific) according to the manufacturer's instructions. The cells were tested for *Mycoplasma* using the *Mycoplasma* PCR Detection kit (ABM). The cells were grown for no more than 15 passages and discarded. Cells were grown in DMEM or DMEM/F12 medium supplemented with 10% FBS. For stable expression of cPD-L1, the cDNA of cPD-L1 (Sino Biological) was inserted into the pGIPZ vector (Horizen Discovery) as described previously (37). Using a pGIPZ-shPD-L1/Flag-cPD-L1 dual-expression construct to knockdown endogenous human PD-L1 and reconstitute Flag-cPD-L1 simultaneously (38), we established endogenous PD-L1 knockdown and Flag-cPD-L1–expressing BT549 cell lines. Lentivirus was packaged by cotransfecting transfer plasmids with pMD2.G (Addgene #12259) and pCMV dR8.2 (Addgene #12263) to HEK293FT cells with X-tremeGENE HP (Roche Diagnostics), and the supernatant was harvested for lentiviral transduction. Selection with 1 μg/mL puromycin (InvivoGen) was routinely performed to maintain ectopic gene expression. For mouse PD-L1 (mPD-L1) knockout, we transfected mPD-L1 double nickase plasmid (Santa Cruz Biotechnology) into MB49 cells using X-tremeGENE transfection reagent. For cPD-L1 overexpression in MB49 cells (MB49^cPDL1^), we infected mPD-L1 knockout MB49 cells with lentivirus carrying pGIPZ-Flag-cPD-L1 followed by selection with puromycin.

### Creation and Selection of Anti-cPD-L1 mAbs

**FIGURE 2:**
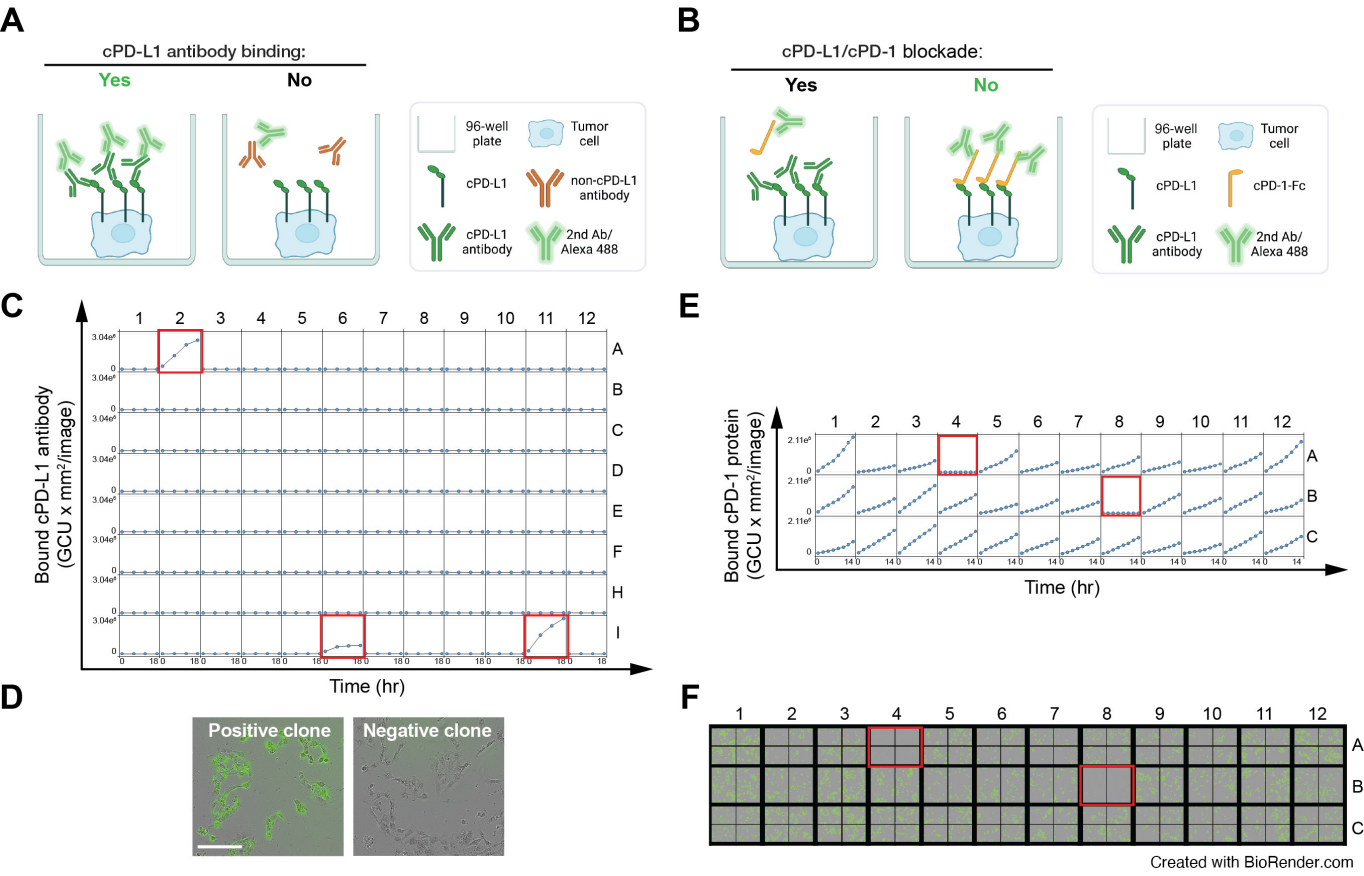
The high-throughput screening of therapeutic antibodies. **A,** Schematic diagram of the cPD-L1 antibody binding assay. BT549 cells expressing cPD-L1 were seeded on 96-well or 384-well plates. cPD-L1 antibodies (from hybridomas) and Alexa Fluor 488–conjugated anti-mouse IgG Fc-specific secondary antibody were added, and then green fluorescence signal was measured to quantify the amount of bound PD-L1 antibody by IncuCyte S3. **B,** A representative result of the cPD-L1 antibody binding assay. Kinetic graphs from each well of a 96-well plate showing quantitative binding of cPD-L1 antibodies on BT549 cells expressing cPD-L1 at 6-hour time intervals. The positive clones are highlighted in red (A2, I6, and I11). **C,** Representative images (at 18 hours) of cPD-L1 antibody binding. Green fluorescent merged images of cPD-L1–expressing cells are shown. **D,** Schematic diagram of the cPD-L1/cPD-1 blockade assay. BT549 cells expressing cPD-L1 were seeded on 96-well or 384-well plates. cPD-1-human IgG Fc (hFc) protein, Alexa Fluor 488–conjugated anti-human IgG Fc-specific secondary antibody and/or cPD-L1 antibody were added, and then green fluorescence signal was measured to quantify the amount of bound PD-1 protein by IncuCyte S3. **E,** A representative result of the cPD-L1/cPD-1 blockade assay. Kinetic graphs from each well of a 96-well plate showing quantitative binding of cPD-1 protein on BT549 cells expressing cPD-L1 at 3-hour intervals after the addition of cPD-L1 antibodies. The positive clones that blocked the interaction of cPD-L1/cPD-1 proteins are highlighted in red (A4 and B8). **F,** Representative images (at 18 hours) of the cPD-L1/cPD-1 blockade. Green fluorescent merged images of cPD-L1–expressing cells are shown. Note the lack of fluorescence due to the antibody binding to PD-L1 and blocking the interaction with cPD-1.

mAbs were generated via conventional hybridoma procedures using A/J mice immunized with the extracellular domain of cPD-L1 (attached to a human Fc tag) at the Vanderbilt University Antibody and Protein Resource Core Facility. Splenocytes were isolated from the immunized mice and then fused with SP2/0 myeloma cells (see Fig. 1). Supernatants from isolated clones were screened for the ability to block the cPD-1/cPD-L1 interaction through cPD-L1–expressing cell-based ELISAs (see Fig. 2A and B for details), with the mAbs 3C8D3 (3C) and 12C10E4 (12C) selected for further study. Clonal antibodies were purified from supernatants and the same assays were repeated.

### Generation of the Canine *CD274* Knock-in Mouse

The caninized PD-L1 mouse (canine *CD274* knock-in mouse) was generated by *Easi*-CRISPR (Efficient additions with ssDNA insert-CRISPR) strategy using a long single-strand DNA (ssDNA) donor and CRISPR ribonucleoproteins (39). Briefly, the long ssDNA (a full-length of canine *CD274* cDNA; NM_001291972) was injected with preassembled guide RNA (gRNA, CAGCAAATATCCTCATGTTT TGG) and Cas9 ribonucleoprotein (ctRNP) complexes into mouse zygotes. The ssDNA and single-guide RNA were synthesized at Integrated DNA Technologies. C57BL/6N female mice at 4 weeks of age (Envigo) were superovulated and then mouse zygotes were obtained by mating C57BL/6N males with the superovulated females. Pronuclei of one-cell stage fertilized mouse embryos were injected with 20 ng/μL Cas9 protein, 10 ng/μL sgRNA, and 5 ng/μL ssDNA. Microinjections and mouse transgenesis were performed as described previously (40). Mouse genomic DNA was extracted from the tail tip and then used for the genotyping (Primer set 1 forward, 5′-CCACTTGGTTCTACATGGCT-3′; Primer set 1 reverse, 5′-CCTCAGCCTGACACATTAGTT-3′; Primer set 2 forward, 5′-CCTGTCACCTCTGAACATGAA-3′; Primer set 2 reverse, 5′-GGACTAAGCTCTAGGTTGTCC-3′; Primer set 3 forward, 5′-GACTGGCTTTTAGGGCTTATGT-3′; Primer set 3 reverse, 5′-ACACCCCACAAATTACTTCCATT-3′) and sequencing (Primer set 3 forward, 5′-GACTGGCTTTTAGGGCTTATGT-3′; Primer set 3 reverse, 5′-ACACCCCACAAATTACTTCCATT-3′) to verify the location of insertion and DNA sequence of canine *CD274*.

### Mouse Study and Antibody Treatment

MB49 ^cPD-L1^ [2 × 10^5^ cells in 25 μL of medium mixed with 25 μL of Matrigel Basement Membrane Matrix (BD Biosciences)] were injected into the flank of the caninized PD-L1 mice (C57BL/6 strain; 6 to 8 weeks old). Mice were divided according to the mean tumor volume in each group. For treatment with antibodies, 100 μg of cPD-L1 antibody (12C10E4 or 3C8D3 clone) or control mouse IgG (Bio X Cell) was injected intraperitoneally on days 4, 6, 8, 10, and 12 after tumor cell inoculation when tumor size was approximately 30 to 40 mm^3^. Tumors were measured every other day with a caliper, and tumor volume was calculated using the following formula: π/6 × length × width^2^.

### Immunofluorescence Study of Mouse Tumor Tissues

Tumor masses were frozen in optimal cutting temperature blocks immediately after excision. Cryostat sections of 5-μm thickness were attached to saline-coated slides. Cryostat sections were fixed with 4% paraformaldehyde for 30 minutes at room temperature and blocked with blocking solution (1% BSA, 2% donkey and/or chicken serum, and 0.1 mol/L PBS) at room temperature for 30 minutes. Samples were stained with primary antibodies against CD8 and granzyme B overnight at 4°C, followed by secondary antibodies at room temperature for 1 hour. Nuclear staining was performed with Hoechst 33342 (Thermo Fisher Scientific). The stained sections were visualized by automated microscopy (Lionheart LX; BioTek Instruments, Inc.). Granzyme B–positive area and the number of CD8-positive CTL were assessed per high power field (200X). Fourteen randomly chosen microscope fields from four serial sections in each tissue block were examined for the number of CD8-positive CTL and granzyme B–positive areas for each tissue.

### Expression and Purification of a Recombinant cPD-L1 Antibody, 12C10E4

The codon optimized for CHO variable light (VL) and heavy (VH) chains were cloned into pTRIOZ-hIgG1 vector (InvivoGen), and then the constant light and heavy chains were replaced with canine kappa light constant chain and canine IgG2 heavy constant chain: pTRIOZ-cIgG2-12C10E4. Plasmids encoding 12C10E4 chimeric antibody, pTRIOZ cIgG2 12C10E4, were transfected into ExpiCHO-S cells following the transfection kit instructions (GIBCO, A29133). ExpiCHO-S cells were cultured with ExpiCHO Expression Medium (Thermo Fisher Scientific) in a shaker incubator set at 120 rpm, 37°C and 8.0% CO_2_. Cells were collected 10 days posttransfection by centrifugation at 4,000 × *g* and 4°C for 20 minutes. The antibody supernatant was passed through a 0.22-μm filter and neutralized with 10XPBS buffer [Lonza BioWhittaker PBS (10X), BW17-517Q] and preincubated with protein A agarose for 2 hours. The agarose A-conjugated antibodies were applied to the column (Bio-Rad poly-prep chromatography column, #731-1550). The column was washed with low-endotoxin PBS [Lonza BioWhittaker Dulbecco's PBS (1X) w/o Calcium and Magnesium, BW17512F24]. Bound antibody was eluted with elution buffer (Thermo Fisher Scientific, Elution Buffers, 0.1 mol/L Glycin-HCl, pH2.8, #21004) into Neutralization Buffer (Tris HCl, 1 mol/L, BP1757-500). Purified antibody was concentrated and buffer exchanged with PBS, pH7.0. The antibody concentration was determined by UV absorbance at 280 nm.

### The Cell-free cPD-L1/cPD-L1 Antibody Binding and cPD-L1/cPD-1 Blockade Assays

ELISA-based assays were performed to compare the receptor-ligand and receptor-antibody binding. The 6X His-tagged extracellular domain of cPD-L1 proteins was expressed in the ExpiCHO cell system (Thermo Fisher Scientific) and purified by the Ni-NTA agarose (Thermo Fisher Scientific) according to the manufacturer's protocol. For the cPD-L1/cPD-L1 antibody binding assay, Pierce Ni-NTA–coated 96-well plates (Thermo Fisher Scientific) were coated with cPD-L1-His protein and 12C10E4 antibody, and anti-canine IgG-specific horseradish peroxidase (HRP)-conjugated secondary antibodies (SouthernBiotech) were added. The bound PD-L1 antibody was quantified by measuring OD_450_ value with a Synergy LX multi-mode reader. For the cPD-L1/cPD-1 blockade assays, Pierce Ni-NTA–coated 96-well plates (Thermo Fisher Scientific) were coated with cPD-L1-His protein and cPD-1-hFc protein (human Fc protein conjugated; SinoBiological US), and anti-human IgG Fc-specific HRP-conjugated secondary antibodies (Thermo Fisher Scientific) were added. Then the cPD-L1 12C10E4 antibody was added. The bound PD-1-Fc protein was quantified by measuring OD_450_ value with a Synergy LX multi-mode reader.

### The Cell Base cPD-L1/cPD-L1 Antibody Binding and cPD-L1/cPD-1 Blockade Assays

The antibody binding and blockade assays were performed as described previously (41). Briefly, to measure PD-L1 protein and PD-L1 antibody interaction, we seeded 1 × 10^4^ BT549^cPD-L1^ cells per well in 96-well plates and then incubated the plates with cIgG control (Rockland Immunochemicals), or 12C10E4 antibody, and anti-canine Alexa Fluor 488 dye conjugate (SouthernBiotech). Every hour, green fluorescent signal was measured and quantified by IncuCyte S3 (Sartorius). To measure PD-1 protein on the cells, we seeded 1 × 10^4^ BT549^cPD-L1^ cells per well in 96-well plates, and then incubated the plates with cIgG control (Rockland Immunochemicals), or 12C10E4 antibody, cPD-1-hFc protein (human Fc protein conjugated; SinoBiological US), and/or anti-human Alexa Fluor 488 dye conjugate (Thermo Fisher Scientific). Every 3 hours, green fluorescent signal was measured and quantified by IncuCyte S3 (Sartorius). The Image analysis was performed according to the manufacturer's protocol.

### Flow Cytometry Analysis

MB49, MB49^cPD-L1^, K9TCC, or K9TCC^nRFP^ cells were washed twice with ice-cold cell staining buffer (BioLegend) and stained with cIgG control or 12C10E4 cIgG for 1 hour at 4°C. After three washes with staining buffer, cell samples were stained with Alexa Fluor 488-conjugated anti-canine IgG-specific secondary antibody for 30 minutes at 4°C. Cell samples were loaded on BD LSRFortessa (BD) for analysis. Data analysis was performed on FlowJo v9 software (BD).

### Binding Affinity (K_D_) Determination

The binding affinity (K_D_) of cPD-L1 protein and cPD-L1 antibody (12C10E4) was determined by Octet Biolayer interferometry using the Octet RED384 system (Sartorius). Briefly, His-tagged cPD-L1 protein was loaded on the Octet NTA biosensor at a concentration of 200 nmol/L. The association step was performed by submerging the sensors in three concentrations of the 12C10E4 antibody (50, 100, 200 nmol/L) in the kinetic buffer. Dissociation was performed and monitored in fresh kinetic buffer. Data were analyzed with Octet Analysis HT software (Sartorius).

### SDS-PAGE and Isoelectric Focusing

The purity and isoelectric point (pI) of the purified antibodies were determined by SDS-PAGE and isoelectric focusing (IEF), respectively. SDS-PAGE or IEF gels were purchased from Bio-Rad Laboratories or Thermo Fisher Scientific. The SDS-PAGE, IEF, and Coomassie blue staining were performed according to the manufacturer's protocol. Image acquisition and quantitation of band intensity were performed using Odyssey CLx infrared imaging system (LI-COR Biosciences).

### Size Exclusion Chromatography

Size exclusion chromatography (SEC) analysis was performed to detect antibody aggregates and monomers. The AKTA Pure 150 M (Cytiva) and Superdex 200 Increase 10/300 GL column (Cytiva) were used to analyze antibodies at a flow rate 0.3 mL/minute for 135 minutes. Elution was monitored using UV absorption at 280 nm, and data were processed by Unicorn 7 software (Cytiva). The SEC analysis was performed in the Molecular Evolution, Protein Engineering, and Production core facility at Purdue University (West Lafayette, IN).

### Peptide Mapping Analysis

The peptide mapping comparison for each 12C10E4 batch was performed. Briefly, the antibody was enzymatically digested with trypsin on S-trap micro columns from Protifi after reduction and alkylation (42). Peptides were then separated and analyzed by a reversed-phase liquid chromatography tandem mass spectrometry (RP-LC/MS-MS) using a Q Exactive HF Hybrid Quadrupole-Orbitrap MS (Thermo Fisher Scientific) equipped with a Nanospray Flex Ion Source (Thermo Fisher Scientific), coupled with a Dionex UltiMate 3000 RSLC Nano System (Thermo Fisher Scientific). The resultant mass spectrometric data were analyzed using the PEAK PTM workflow in the PEAKS X PRO Studio 10.6 software package from Bioinformatics solutions Inc. to map the detected MS1 and MS2 ions to the amino acid sequence of antibody (43). The peptide mapping analysis was performed in the Bindley Bioscience Center Purdue Proteomics Facility at Purdue University (West Lafayette, IN). LC/MS-MS data were used for mapping glycosylation (0.98 Da) of asparagine (N) and glutamine (Q) residues of the mapped antibody sequences.

### N-Glycomic Analysis of 12C10E4 Antibody

*N*-glycomic analysis of 12C10E4 antibody was performed by methods described previously (44). Briefly, *N*-glycans of 12C10E4 antibody were released by treating the reduced and alkylated protein with PNGase F. The released *N*-glycan fractions were then permethylated. The permethylated *N*-glycans were evaluated by Matrix-assisted laser desorption/ ionization – mass spectrometry (MALDI-MS) using the AB SCIEX TOF/TOF 5800 mass spectrometer (Applied Biosystem/MDS Analytical Technologies). The structural assignments of the *N*-glycans were based on molecular weight and followed the principles of the *N*-glycan biosynthesis pathway. The carbohydrate analysis was performed at the Complex Carbohydrate Research Center, the University of Georgia (Athens, GA; supported by NIH R24GM137782 grant).

### Activation of Canine PBMCs and Cytokine Measurement

Primary canine PBMCs (cPBMC) were isolated from dog blood using SepMate PBMC isolation tubes (Stemcell Technologies) and Histopaque-1077 (Millipore Sigma) per the manufacturer's protocol. The activation of canine T cells by anti-canine CD3 and CD28 antibodies has been well established in the previous studies (45–47). Briefly, the cPBMCs were activated with 10 ng/mL canine IL2 (Novus Biologicals), and cotreated with and without 1 μg/mL anti-canine CD3ε antibody (CA17.2A12 clone, coated; Thermo Fisher Scientific), and 3 μg/mL anti-canine CD28 antibody (1C6 clone; Thermo Fisher Scientific) for 48 hours. MILLIPLEX Canine Cytokine/Chemokine Magnetic Bead Panel (Millipore) was used to multiplex and measure IFNγ, IL10, and TNFα in these activated canine PBMCs following manufacturer's protocols. Samples were incubated with the cytokine magnetic beads on shaker for 2 hours followed by incubation with secondary detection antibody provided in the kit. The plate was read on an Attune flow cytometer (Thermo Fisher Scientific) by using the FL2 (PE channel) channel for the reporter and FL4 (APC channel) for classification. For each of the cytokines, 300 beads were measured, and data were collected as a forward and side scatter dot plot. Concentrations of cytokines were quantified as ng/mL using Cytokine Multiplex Analysis Software (MPLEX, Cytomics Analytical LLC). The data show a significant induction in the expression of IFNγ, IL10, and TNFα with anti-CD3/CD28 and IL2 treatment as compared with IL2 treatment alone, and this activation protocol was used for the remainder of the work.

### NanoString Analysis of Activated cPBMCs

RNA was isolated from activated cPBMCs as described previously (RNeasy kit, Qiagen) and submitted to the Stark Neurosciences Research Institute Biomarker Core, Indiana University School of Medicine, Indiana University, Indianapolis, IN, for detection of modulation of genes upon activation using the nCounter Canine IO Panel (NanoString Technologies). Data were analyzed using Rosalind (Rosalind). Groupwise comparison was conducted using control cPBMCs and compared with activated cells from three dogs. Differentially expressed genes (Fold change (FC) ≥ 1.5; *P* < 0.05) were considered significant. Data were visualized using heatmap, volcano plot, and histogram for specific genes.

### Tumor Cell Killing Assay

The tumor cell killing assay was performed according to the previous description (48). To analyze the killing of tumor cells by cPBMCs, nuclear-restricted red fluorescent protein (RFP)-expressing K9TCC cells were cocultured with activated cPBMCs cells in DMEM/F12 with 10% FBS. cPBMCs were activated by incubation with 100 ng/mL anti-canine CD3ε antibody (CA17.2A12 clone, Thermo Fisher Scientific), and 10 ng/mL canine IL2 (Novus Biologicals) in DMEM/F12 with 10% FBS. After 96 hours, RFP signals were measured as surviving tumor cells, and the expression of IFNγ, IL10, and TNFα in the supernatant of the cocultured cells was measured by MILLIPLEX Canine Cytokine/Chemokine Magnetic Bead Panel according to the manufacturer's protocols.

### Pilot Study in Laboratory Dogs

A single-dose pilot study to assess initial safety and pharmacokinetic parameters was performed in six laboratory beagles approximately 12–15 months old, with male and female dogs included. The dogs were housed and evaluated in the Pre-Clinical Research Laboratory, College of Veterinary Medicine, Purdue University, West Lafayette, IN. The 12C cPD-L1 chimera antibody for the laboratory dog study was produced in the Molecular Evolution, Protein Engineering, and Production Facility at Purdue University (West Lafayette, IN). The antibody solution was *Mycoplasma* free and contained <0.5 EU endotoxin/mg antibody (consistent with the endotoxin limit for human PD-L1 antibody solutions). After being acclimated to the facility, the dogs were treated with the 12C cPD-L1 chimera antibody diluted in sterile PBS for intravenous administration (6 mL/kg body weight total volume) and administered through an intravenous catheter over 1 hour. Six dogs were treated and received 2 or 5 mg/kg antibody. Blood was collected for pharmacokinetic analyses prior to treatment and at 1, 6, 24, 48, and 72 hours after the start of the antibody administration, and then once weekly for 4 weeks. Monitoring for adverse events included physical exam before and during treatment, then twice daily for 7 days, and then weekly for 4 weeks; daily observation for 4 weeks; and a complete blood count (CBC), serum biochemistry panel, and urinalysis before treatment and weekly for 4 weeks. Additional tests specific to any adverse events observed could be added. Adverse events were categorized using Veterinary Cooperative Oncology Group (VCOG) criteria [Veterinary Cooperative Oncology Group – common terminology criteria for adverse events (VCOG-CTCAE) following chemotherapy or biological antineoplastic therapy in dogs and cats v1.1] (49).

### Detection of the cPD-L1 Antibody in Dog Serum

The concentration of cPD-L1 antibody in dog serum was determined in pharmacokinetic analysis. Pierce Ni-NTA–coated 96-well plates (Thermo Fisher Scientific) were coated with cPD-L1-His protein and serially diluted dog serum (1/100, 1/200, and 1/400), and anti-canine IgG-specific HRP-conjugated secondary antibodies (SouthernBiotech) were added. The standard curve was obtained from the standard samples of 12C10E4 antibodies prepared in standard sample dilution buffer: 0, 7.81, 15.63, 31.25, 62.5, 125, 250 ng/mL. The bound PD-L1 antibody was quantified by measuring OD_450_ value with a Synergy LX multi-mode reader.

### Statistical Analysis

All quantitative results were displayed as the mean ± SD, with at least three biological replicates. The intergroup statistical significance was calculated by two-tail Student *t* test. *P* < 0.05 was considered statistically significant.

### Data Availability Statement

The data generated in this study are available upon request from the corresponding author.

## Results

### Development and High-throughput Screening of Anti-cPD-L1 Antibodies

Anti-cPD-L1 mAbs were successfully generated using conventional hybridoma procedures (50). Please see Fig. 1, which summarizes an overview of cPD-L1 antibody development. Antibodies were screened using PD-L1 binding and blockade assays similar to those we described previously (refs. 37, 41, 48; Fig. 2A and B). Among over 2,000 hybridomas, 154 clones were selected against membrane-localized cPD-L1 protein in a live cell–based antibody binding assay (Fig. 2C and D). Of these, 10 clones were found to block the cPD-L1/cPD-1 interactions (Fig. 2E and F). Representative positive clones are shown in Fig. 2B–F. On the basis of the specificity, binding affinity, and PD-1/PD-L1 blockade efficacy, we selected the antibodies termed 3C8D3 (3C) and 12C10E4 (12C) for further analysis.

### Evaluation of Therapeutic Efficacy of cPD-L1 Antibodies in the Caninized PD-L1 Mice

**FIGURE 3:**
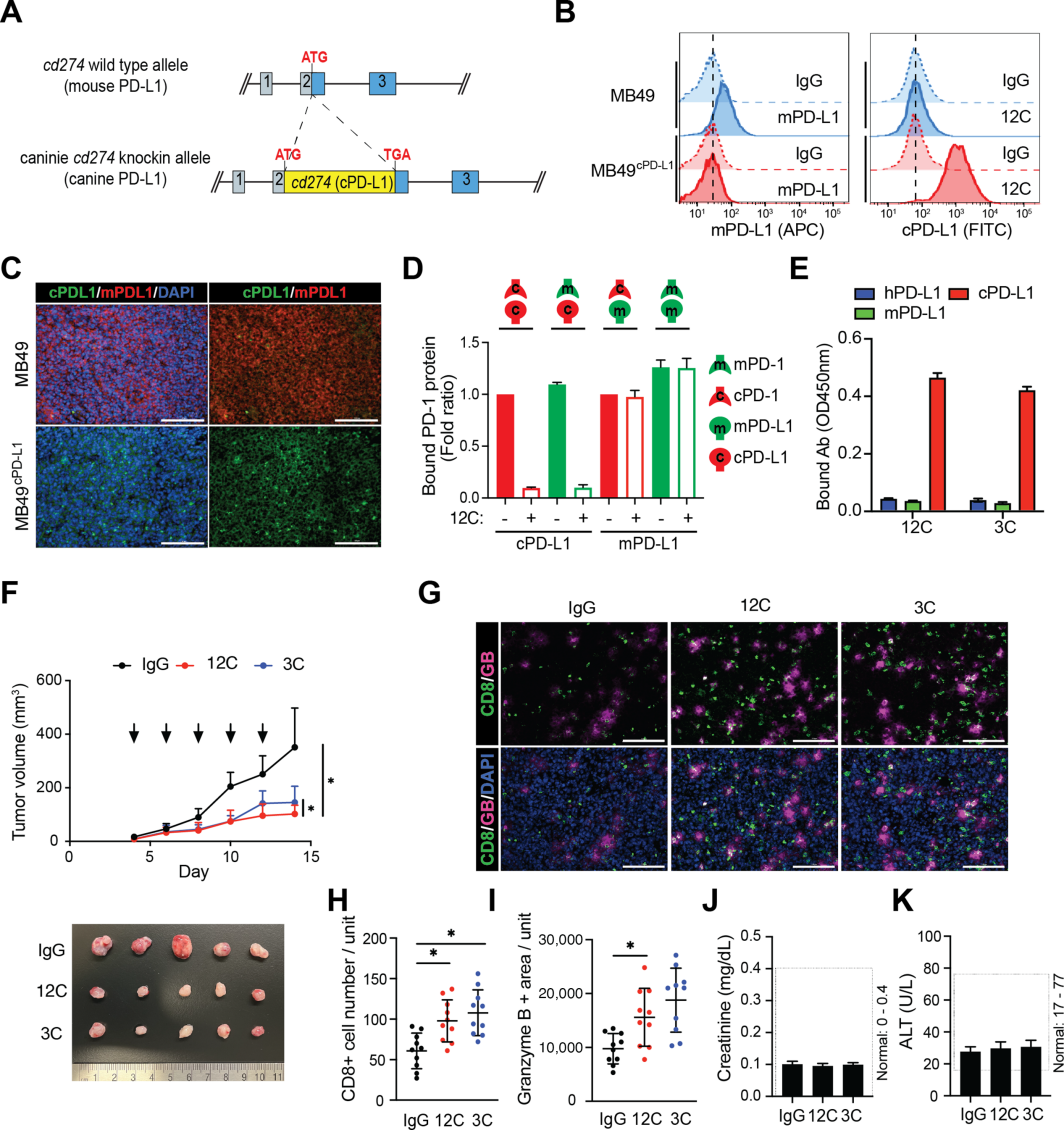
cPD-L1 antibodies enhance antitumor immunity in the caninized PD-L1 syngeneic mouse model. **A,** Knock-in strategy of the caninized PD-L1 mice (c57BL/c background). **B,** Validation of cPD-L1 protein expression in the MB49^cPD-L1^ cells. Flow cytometric analysis of membrane located mPD-L1 and cPD-L1 protein in MB49 cells expressing cPD-L1 (MB49^cPD-L1^) or MB49 parental cells. **C,** Immunofluorescence staining and protein expression pattern of mPD-L1 and cPD-L1 in MB49 or MB49^cPD-L1^ tumor masses from the caninized PD-L1 mice. DAPI, nuclear counterstaining. Scale bar, 100 μm. **D,** Interaction of cPD-1 or mPD-1 protein with cPD-L1 or mPD-L1 protein with or without cPD-L1 antibody, 12C. His-tagged canine or mPD-L1 protein was immobilized on the Ni-NTA 96-well plate, and HRP-conjugated anti-human IgG Fc-specific secondary with mPD-1-hFc or cPD-1-hFc protein was added. OD_450_ was measured to quantify the amount of bound PD-1 protein. **E,** Binding of cPD-L1 antibodies, 12C and 3C, with human PD-L1 (hPD-L1), mPD-L1, and cPD-L1 proteins. His-tagged human PD-L1, mPD-L1, or cPD-L1 protein was immobilized on the Ni-NTA 96-well plate, and anti-cPD-L1 antibodies, 12C or 3C, with HRP-conjugated anti-canine IgG-specific secondary was added. OD_450_ was measured to quantify the amount of bound PD-L1 antibodies. Ab, antibody. **F,** Tumor growth of MB49^cPD-L1^ in the caninized PD-L1 mice treated with cPD-L1 antibody, 12C or 3C. The IgG isotype of 12C and 3C antibodies is mouse IgG1 which is equivalent to human IgG4. Tumors were measured at the indicated timepoints (*n* = 8 per group). At the endpoint, the tumors were dissected. **G–I,** Immunofluorescence staining, and protein expression pattern of CD8 and granzyme B in MB49 tumor masses from IgG-, 12C-, or 3C-treated mice. DAPI, nuclear counterstaining. Scale bar, 100 μm. Representative images of immunostaining of CD8 and granzyme B in the MB49 tumor mass (G). CD8 (H) and granzyme B (I) were quantified using Gen5 software (BioTek). *n* = 10. Treatment with the PD-L1 antibody did not affect kidney function (serum creatinine; **J**) or liver enzyme activity (ALT; **K**), measured in blood collected at the end of the experiment. ALT, alanine aminotransferase.

To further assess the clinical use of the cPD-L1 antibody as an immunotherapeutic drug, its therapeutic efficacy needed to be evaluated in an appropriate *in vivo* model. To do so, we established caninized PD-L1 (C57BL/6 background) mice. Briefly, we generated mice that expressed cPD-L1 on the cell surface by replacing the mouse *Cd274* with canine *CD274* using a CRISPR knock-in mouse strategy (Fig. 3A). To evaluate the therapeutic efficacy of the cPD-L1 antibodies in a syngeneic animal model, we generated the mouse bladder cancer cell line MB49-expressing cPD-L1 (MB49^cPD-L1^) by knocking out mPD-L1 and reexpressing cPD-L1 (Fig. 3B and C). Although MB49^cPD-L1^ and the caninized PD-L1 mice express cPD-L1 protein instead of mPD-L1 protein, these caninized PD-L1 mice express mPD-1 protein. Therefore, we examined whether cPD-L1 protein interacts with mPD-1 protein before evaluating the therapeutic efficacy of the cPD-L1 antibody in the caninized PD-L1 mice. The binding of cPD-L1 and mPD-1 was similar to the cognate cPD-L1 and cPD-1 pair (Fig. 3D). Consistently, the cPD-L1 antibody (12C) efficiently blocked both the cPD-L1/mPD-1 and cPD-L1/cPD-1 interactions, but not that of mPD-L1/mPD-1 or mPD-L1/cPD-1 as the cPD-L1 antibodies do not recognize mPD-L1 (Fig. 3D and E).

Treatment of MB49^cPD-L1^ tumors in the cPD-L1 mice with either the 12C or 3C antibody significantly reduced the tumor size (Fig. 3F), and increased the number of infiltrating cytotoxic T cells relative to mice treated with control IgG as measured by CD8 and granzyme B expression (Fig. 3G–I). Both the 12C and 3C antibodies demonstrated good safety profiles in mice in that body weight was maintained, and there were no changes in kidney function as assessed by serum creatinine or liver enzyme activity (Fig. 3J and K). The *in vitro* and *in vivo* validation results indicated that the cPD-L1 antibodies that recognize cPD-L1 effectively inhibit the PD-1/PD-L1 pathway and enhance mouse antitumor immunity.

### Characterization and Evaluation of the Caninized cPD-L1 Chimeric Antibody as a New Immunotherapeutic Antibody

**FIGURE 4:**
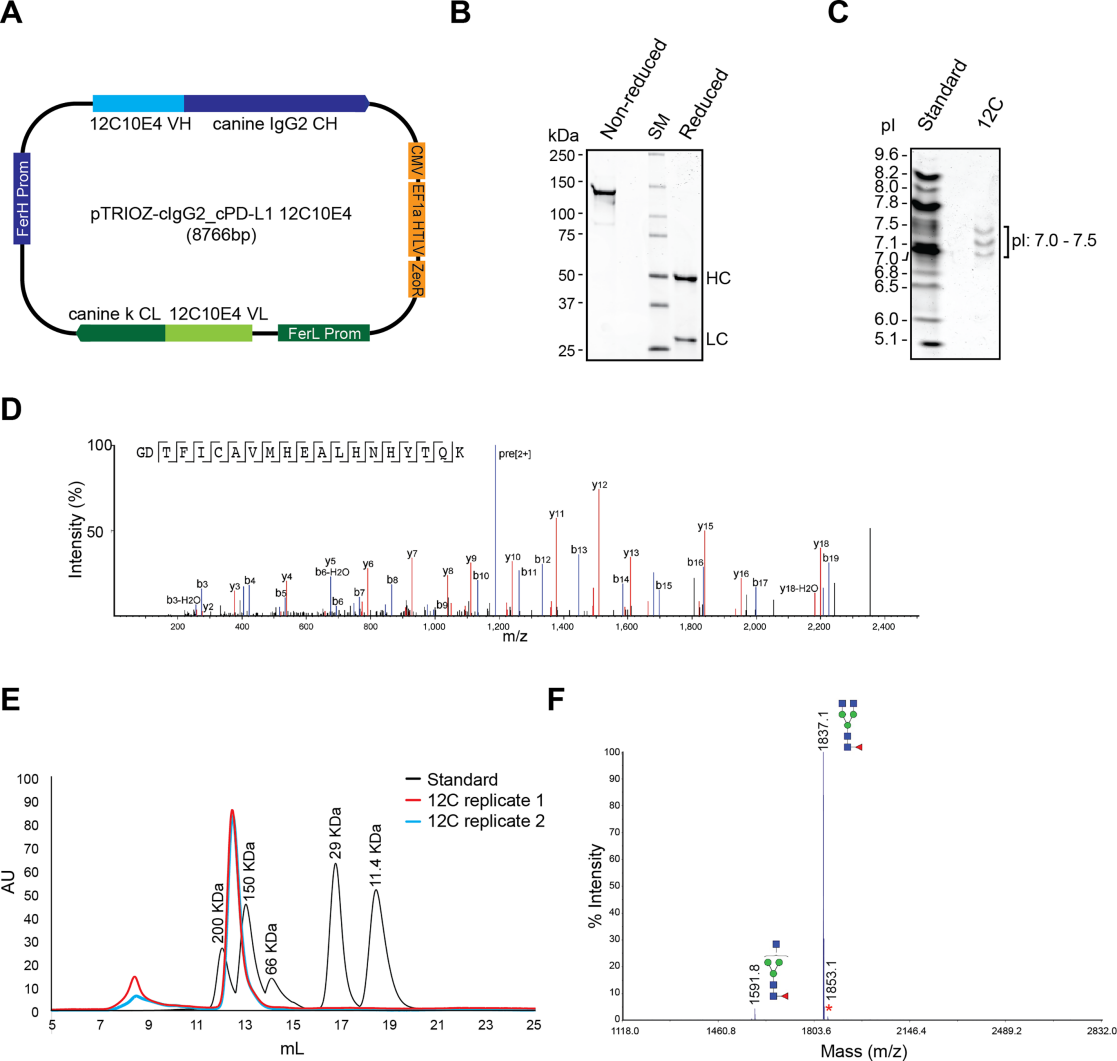
The quality attributes of the purified anti-cPD-L1, 1210E4 (12C) chimeric antibody. **A,** Canine PD-L1, 12C10E4 chimeric antibody expression construct, pTRIOZ-cIgG2-cPD-L1 12C10E4. **B,** SDS-PAGE analysis of 12C chimeric antibody purity under nonreducing and reducing (2-mercaptoethanol) conditions. HC, heavy chain; LC, light chain; SM, protein size marker. **C,**IEF analysis of 12C chimeric antibody. Standard, pI standard. **D,** Peptide mapping analysis. The peptide mapping of cPD-L1 12C chimeric antibody. The 12C chimeric antibody was enzymatically digested with trypsin on S-trap micro columns from Protifi after reduction and alkylation. Peptides were then separated and analyzed by RP-LC/MS-MS. The resultant mass spectrometric data were analyzed using the PEAK PTM workflow in the PEAKS X PRO Studio 10.6 software package from Bioinformatics solutions Inc. to map the detected MS1 and MS2 ions to the amino acid sequence of antibody. The sequence coverage of heavy and light chains was 100% (453 of 453 amino acids) and 98.2 (223 of 227 amino acids), respectively. **E,**SEC analysis of 12C chimeric antibody. Standard, SEC standard. **F,** MALDI-MS profiling of permethylated N-glycans released from PNGase F-treated 12C chimeric antibody. The masses of indicated glycan species represent the [M + Na+] values.

For clinical use of cPD-L1 antibodies in dogs, the 12C antibody was caninized by replacing the mouse constant domain with canine IgG2 (equivalent to human IgG1) constant domains. Briefly, we sequenced full-length VH and VL RNA transcripts obtained from hybridoma clones by 5′/3′ RACE and cloned these into the pTRIOZ-cIgG2-ck vector, which is designed for high-yield production of whole mAbs from a single plasmid (Fig. 4A). The chimeric cPD-L1 antibody retained the cPD-L1 binding VH and VL chains of the mouse hybridoma.

**FIGURE 5:**
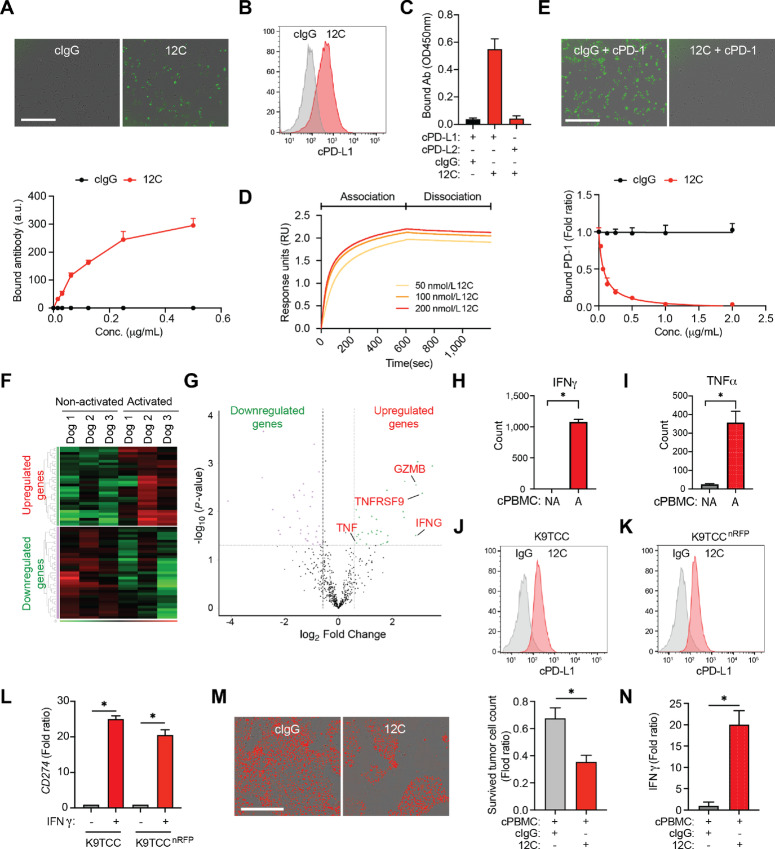
Evaluation of the caninized cPD-L1 chimeric antibody. **A,** 12C antibody binding on the BT549^cPD-L1^ cells. **B,** Flow cytometric analysis of the 12C chimeric antibody on the BT549^cPD-L1^ cells. cIgG serves as a negative control. **C,** 12C chimeric antibody binding to cPD-L1 and cPD-L2. **D,** Binding affinity (K_D_) analysis of 12C chimeric antibody by Octet. **E,** EC_50_ of 12C chimeric antibody, 12C10E4. EC_50_ = 0.419 μg/mL. The bound cPD-1 protein was quantified by measuring green fluorescence at the IncuCyte S3. **F** and **G,** Canine IO Panel (NanoString) analyses were used to query changes in gene expression upon activation of cPBMCs from three healthy pet dogs. The RNA from the resting and activated PBMCs was used for NanoString work. The Canine IO panel was used to query the changes in approximately 700 genes. Groupwise analyses were conducted using “Rosalind.” There were 65 genes that were differentially expressed when comparing control PBMCs with activated PBMCs (*P* < 0.05, FC > 1.5) including 30 upregulated and 35 downregulated genes. In the heatmap, each column consists of data from one sample. IFNγ (**H**) and TNFα (**I**) concentrations were analyzed in the activated canine PBMCs. **J** and **K,** Flow cytometric analysis of cPD-L1 protein expression on the K9TCC or the nuclear-restricted RFP-expressing K9TCC (K9TCC^nRFP^) cells using the 12C chimeric antibody. The endogenous PD-L1 expression was stimulated by 50 ng/mL canine IFNγ for 12 hours. cIgG served as a negative control. **L,** The quantitative RT-PCR analysis of cPD-L1 (*CD274*) mRNA expression in the K9TCC or K9TCC^nRFP^ cells. **M,** The 12C chimeric antibody enhances the tumor cell killing. Canine bladder cancer, K9TCC cells were cocultured with cPBMCs that were activated with CD3 antibody (100 ng/mL) and IL2 (10 ng/mL) at a ratio of 1 tumor cell: 15 cPBMCs. The live tumor cell count at 72 hours is shown in the bar graph. **N,** IFNγ concentrations were analyzed in the medium from the coculture of the K9TCC cells and activated cPBMCs with/without the 12C chimeric antibody treatment.

To monitor batches during antibody production, we identified the attributes of the purified chimeric antibodies, such as purity, pI value, amino acid sequence, and N-glycomic profile (Fig. 4B–F). The chimeric antibody, 12C10E4-cIgG, bound to the membrane-localized cPD-L1 protein (Fig. 5A and B), but did not recognize cPD-L2 protein (Fig. 5C). The affinity (K_D_) of the chimeric antibody as determined by Octet was 8.6 nmol/L (Fig. 5D). Similar to the murine 12C antibody obtained from the hybridoma, the 12C chimeric antibody blocked the cPD-L1/cPD-1 interaction (EC_50_ = 0.419 μg/mL; Fig. 5E).

To demonstrate immune checkpoint inhibition of the 12C chimeric antibody, an *ex vivo* canine system such as a tumor cell killing assay in which cPD-L1–positive canine bladder cancer cells (K9TCC) are cocultured with activated canine PBMCs was established. The canine immune-oncology panel analysis (Fig. 5F and G; Supplementary Table S1) and analysis of secreted cytokines (IFNγ and TNFα; Fig. 5H and I) demonstrated the activation of canine PBMCs by anti-canine CD3 and CD28 antibodies and canine IL2 treatment. To quantify the number of surviving or dead tumor cells in a tumor cell killing assay, we established K9TCC^nRFP^ cells expressing nuclear-restricted RFP (nRFP) and confirmed the expression of endogenous cPD-L1 protein and mRNA in both K9TCC parental and K9TCC^nRFP^ cells upon IFNγ treatment (Fig. 5J–L). We used these activated canine PBMCs and K9TCC^nRFP^ cells to perform a tumor cell killing assay. Although the PBMC and tumor cells were from different dogs, and thus the dog lymphocyte antigen (DLA) was not matched between the cPBMCs and K9TCC cells, the 12C chimeric antibody enhanced tumor cell killing activity and IFNγ secretion (Fig. 5M and N).

**FIGURE 6:**
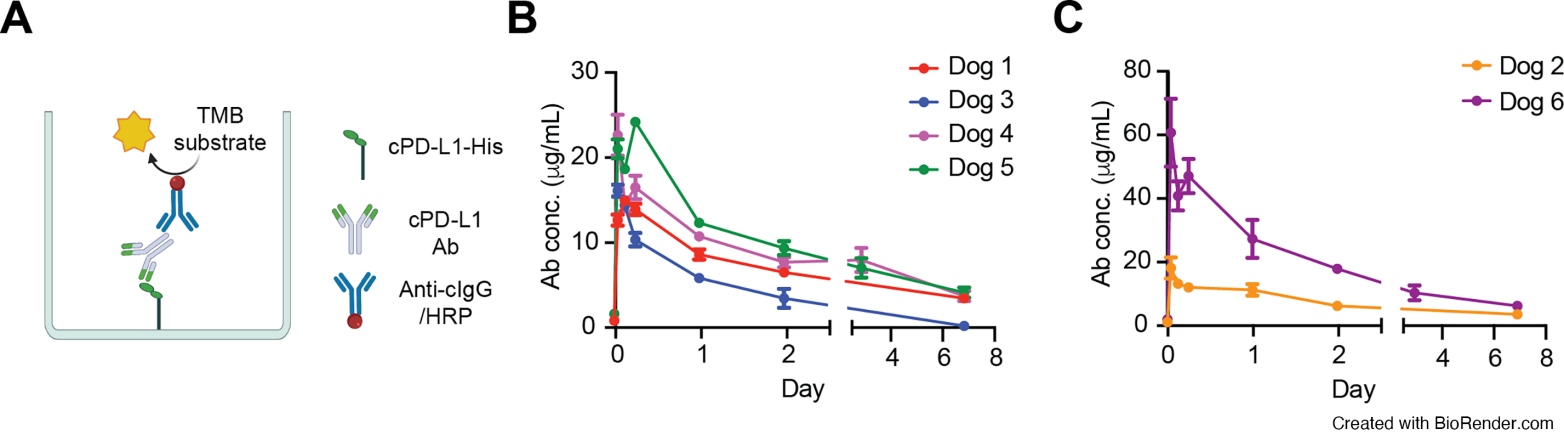
Pharmacokinetic analysis of 12C chimeric antibody in laboratory dogs. **A,** Schematic diagram of ELISA for pharamacokinetic analysis. The concentration of the 12C chimeric antibody was measured in the 2 mg/kg (**B**) or 5 mg/kg (**C**) 12C antibody-treated dogs’ serum.

To study the half-life of the 12C chimeric antibody in dogs, we established a new ELISA-type assay using purified his-tagged cPD-L1 protein (cPD-L1-His) and measured the concentration of 12C chimeric antibody in dog serum (Fig. 6A). In an initial single-dose pharmacology study in beagle dogs, the 12C chimeric antibody was well tolerated and had a half-life of 1 to 2 days (Fig. 6B and C; Table 1). Interestingly, the half-life of the cPD-L1 antibody was shorter than that for human checkpoint inhibitors and indicated that weekly dosing could be appropriate in dogs. The possible infusion reaction in one dog resolved without intervention. There was good antibody tolerability of the antibody in this single-dose study in the lab dogs and body weight was maintained (Table 1). Nonspecific changes such as a slight reduction in monocyte count reduction and a slight increase in CO_2_ and gamma-glutamyl transferase (GGT) were transient and resolved without intervention (Table 1).

**TABLE 1:** Summary of potential adverse events with the initial cPD-L1 antibody administration to laboratory dogs

Parameter	Result
Dogs	3 intact female, 3 intact male beagles, 12–15 months of age
Antibody dosing	Administered 2 mg/kg (4 dogs) or 5 mg/kg (2 dogs) antibody given by slow intravenous infusion over 1 hour
Abnormalities in complete blood counts or serum biochemical profiles	Slightly low monocyte count (0.09 × 10^3^/μL, reference range 0.15–1.35 × 10^3^/μL) at 1 week after treatment; normalized by 2 weeks after treatment (1 dog, 2 mg/kg cPD-L1 antibody). Slightly high CO_2_ (25 mmol/L, reference range 13–24 mmol/L) at 1 week after treatment; normalized by 2 weeks after treatment in 1 dog and by 4 weeks after antibody treatment in a second dog (1 dog, 2 mg/kg and the second dog 5 mg/kg cPDL-1 antibody). Slightly high GGT (18 IU/L, reference range 5–16 IU/L) at 1 week after treatment; normalized by 2 weeks after treatment (1 dog, 5 mg/kg cPD-L1 antibody). Slightly high cholesterol (306 mg/dL, reference range 124–301 mg/dL) at 1 week after treatment; normalize by 6 weeks after treatment (1 dog, 5 mg/kg cPD-L1 antibody).
Other observations	Possible infusion reaction in 1 dog who experienced weakness, pale mucous membranes, and bradycardia (reduced heart rate) that started 5 minutes after the completion of the antibody infusion. The dog returned to normal within 10 minutes with no intervention (1 dog, 5 mg/kg cPD-L1 antibody). Mildly decreased appetite the day of treatment in 3 dogs (2 dogs, 2 mg/kg cPD-L1 antibody; 1 dog 5 mg/kg cPD-L1 antibody). Other than these findings, the dogs remained bright, alert, and active, maintained body weight, and had normal temperature/pulse/respiration.

## Discussion

Companion dogs naturally develop several types of cancer that in many respects resemble clinical cancer in human patients (51). Although mouse models are the most commonly used animal model in cancer research, they do not possess collective features such as tumor heterogeneity, mutational landscape, cancer molecular subtypes, and immune cell responsiveness present in human cancer (7). Therefore, studies in mouse models should be complemented by other models such as specific forms of naturally occurring cancer in pet dogs (51). These complementary studies are especially important for the development or study of new immuno-oncology drugs like novel immune checkpoint inhibitors (ICI) or of combination regimens for companion dogs that can develop naturally occurring cancer in the context of an intact immune system and an aggressive heterogeneous cancer. The development of canine ICIs is expected to expand comparative oncology approaches to improve the current therapeutic efficacy of immunotherapies in human cancer. Therefore, the canine studies of immuno-oncology drugs produce translatable knowledge that can inform and prioritize new immuno-oncology therapy in humans. The challenge has been, however, that ICIs that target canine immune checkpoint molecules such as cPD-1 and cPD-L1 have not been commercially available.

We successfully developed a new cPD-L1 antibody as an immuno-oncology drug and characterized its functional and biological properties using multiple assays including a cPBMC-mediated canine tumor cell killing assay. It is recognized that in this assay (Fig. 5M and N), the increase in tumor cell killing activity associated with the 12C antibody may not directly represent T cell–mediated activity due to an unmatched DLA between cPBMCs and K9TCC cells. However, the PD-L1/PD-1 interaction also plays a role in maintaining allogeneic immune tolerance (52–54). Therefore, the 12C chimeric antibody can inhibit the immunosuppressive function of cPD-L1 in the coculture setting of canine T cells and allogenic bladder cancer cells. Natural killer (NK) cells or monocytes in the PBMCs might also contribute to the cytotoxic activity due to the unmatched DLA between T cell and tumor cells. Given that the expression of PD-L1 on T cells and NK cells has been reported (55, 56), together with the blockade of PD-L1 on cancer cells by the 12C antibody, a direct effect of PD-L1 on T cells or other immune cells may also enhance the efficacy of the 12C antibody in the caninized PD-L1 mice in addition to the targeting of PD-L1 on cancer cells. Results from our evaluation of the therapeutic efficacy of cPD-L1 antibodies in our unique caninized PD-L1 mice prompted us to caninize the cPD-L1 antibody, 12C10E4, for use in dog studies. As a strategy of caninization, we made a chimeric antibody by fusing murine variable regions to canine constant regions. The 12C chimeric antibody retained the functional properties of the original 12C murine antibody produced from the hybridoma. On the basis of the quality attributes of the 12C chimeric antibody (Figs. 4 and 5) and the initial safety profile in the laboratory dogs (Table 1), our cPD-L1 antibody is a promising ICI for dogs. We anticipate that our chimeric cPD-L1 antibody will be an efficacious immune checkpoint blockade antibody for patients with canine cancer.

The positive results from the single-dose pilot study in lab dogs included detection of *in vivo* concentrations of cPD-L1 antibody that had good *in vitro* activity in PD-L1 binding and blockade assays and cPBMC-mediated killing of tumor cells. The shorter half-life of the cPD-L1 antibody compared with human checkpoint inhibitors (i.e., 27 days for atezolizumab, 18 days for durvalumab, 4 days for avelumab; ref. 57), indicated that weekly dosing could be appropriate in dogs. The pharmacokinetic parameters and safety will be confirmed in multi-dose studies in dogs, and antibody occupancy will be assessed in future work in tumor-bearing dogs. Of note, infusion reactions commonly occur in humans treated with checkpoint inhibitors, and management of these reactions can include slowing the rate of infusion and pretreatment with antihistamines in subsequent doses (58). It will be important to continue to monitor treated animals for infusion reactions in multi-dose studies in dogs, as the rate of these reactions could increase with continued exposure to the antibody.

Two groups previously reported studies of canine anti-PD-L1 and anti-PD-1 antibody treatment in dogs with cancer (30, 32). Maekawa and colleagues studied a rat-canine chimeric anti-PD-L1 mAb (c4G12) in dogs with oral malignant melanoma (OMM) and undifferentiated sarcoma, and Igase and colleagues studied rat-canine-chimeric (ch-4F12-E6) and caninized anti-PD-1 (ca-4F12-E6) antibodies in dogs with OMM as well as other spontaneous tumors including squamous cell tumors and cutaneous melanoma (30, 32). Because treatment of canine cancers with anti-PD-L1 or anti-PD-1 antibodies is a fairly unexplored area, there were some limitations in these initial studies such as sample size as well as variation in study cases such as cancer type and stage, and status of prior treatment (i.e., treatment-naïve vs. history of prior treatment), which could have contributed to the inconsistency and variation in outcomes. Nonetheless, both groups demonstrated the potential of these antibodies to treat certain cancer types, especially OMM, with positive responses noted (30, 32). In addition, the mAbs designed by Choi and colleagues further supported the ability of anti-PD-L1 antibodies to augment IFNγ secretion in PBMC cultures that is suggestive of the potential of PD-L1 blockade to reinvigorate T-cell activity in canine tumors (31). Recently, Maekawa and colleagues further studied their c4G12 anti-PD-L1 antibody in a group of dogs with primary OMM and, consistent with responses in human studies, observed good safety with no serious adverse events recorded (33). They did observe antitumor responses in some dogs in the treatment group, and the study outcomes were suggestive of enhanced survival with treatment compared with the control group (33). Despite limitations associated with the nature of the immunotherapy administration for treatment of canine cancers and the relatively recent and novel nature of these treatments, the studies discussed support the clinical promise of antibodies targeting the PD-1/PD-L1 axis and the need for further studies. Considered together with antibodies described by other groups, our new cPD-L1 antibody will broaden treatment options for patients with canine cancer and could provide clinical benefits similar to those offered by the human PD-L1 antibodies, atezolizumab, durvalumab, and avelumab that have undergone extensive clinical assessment.

In the development of immuno-oncology drugs, particularly immunotherapeutic antibodies, translation of discoveries in mouse models to clinical trials has been hindered by multiple biological differences between mice and humans, such as the lack of cross-reactivity between species. For example, if an anti-human or cPD-L1 antibody does not recognize mPD-L1 protein, the therapeutic efficacy of that antibody cannot be evaluated in a syngeneic mouse model. Mice with an engrafted human immune system have been developed for translational research to help overcome this constraint. Indeed, pembrolizumab, an anti-human PD-1 antibody, showed tumor growth inhibition and CD8^+^ T-cell activation in humanized NSG mice that received tumor implants from patient-derived xenografts (59). Despite the importance of tumor-bearing mice with engraftment of a human immune system for preclinical immuno-oncology research, this model nonetheless presents considerable obstacles such as a limited source of human cells and tissues, immune rejection, and high cost (60). As an alternative mouse model for immunotherapeutic antibody development, humanized immune checkpoint mice are commercially available. For example, humanized PD-L1 mice have been generated by replacing the mPD-L1 gene (*Cd274*) with the human PD-L1 gene (*CD274*) using CRISPR/CAS9 methods. The humanized PD-L1 mouse can be used to evaluate the therapeutic efficacy of anti-human PD-L1 antibodies *in vivo*. However, no caninized PD-L1 mouse model has been previously reported. The lack of a suitable mouse model is a major obstacle for developing canine ICIs such as PD-1/PD-L1 blockade antibodies, in which the ideal approach would be to move successful treatment approaches in mice to studies in dogs, and to further translate those with the highest success in dogs into human trials. To overcome this obstacle, we established a caninized PD-L1 mouse model as a preclinical tool, and validated the therapeutic efficacy of immunotherapeutic cPD-L1 antibodies *in vivo* (Fig. 3). Our caninized PD-L1 mouse model and syngeneic mouse bladder cancer cell line, MB49^cPD-L1^, are unique and powerful tools for preclinical canine immuno-oncology research.

In conclusion, our cPD-L1 antibody and unique caninized mouse model will be critical research tools to improve the efficacy of immune checkpoint blockade therapy in both dogs and humans. Furthermore, these tools will open new perspectives for immunotherapy applications in cancer as well as other autoimmune diseases that could benefit a diverse and broader patient population.

## Supplementary Material

Table S1 The list of genes that were altered in the activated canine PBMCs from the nCounter canine immune-oncology panel analysis
