# Supplementary material for: Development of an Anti-canine PD-L1 Antibody and Caninized PD-L1 Mouse Model as Translational Research Tools for the Study of Immunotherapy in Humans
Source: Cancer Res Commun. 2023 May 15;3(5):860–73. doi: 10.1158/2767-9764.CRC-22-0468 (PMC10184575; doi:10.1158/2767-9764.CRC-22-0468)
Supplement: Table S1 — The list of genes that were altered in the activated canine PBMCs from the nCounter canine immune-oncology panel analysis [file crc-23-0468-s01.docx]

**Supplementary information**

**Table S1**. The list of genes that were altered in the activated canine PBMCs from the nCounter canine immune-oncology panel analysis.

| Name | | Description | Fold Change | Log2 Fold Change | p-Value | p-Adj | Ave. Log2 Exp. |
| --- | --- | --- | --- | --- | --- | --- | --- |
| S100A4 | | S100 calcium binding protein A4 | 2.70 | 1.43 | 0.02 | 0.34 | 11.23 |
| IFNG | | interferon gamma | 7.32 | 2.87 | 0.03 | 0.35 | 8.35 |
| IL2RA | | interleukin 2 receptor, alpha | 1.84 | 0.88 | 0.03 | 0.34 | 9.80 |
| TNF | | tumor necrosis factor | 1.60 | 0.67 | 0.04 | 0.39 | 8.56 |
| CSF2 | | colony stimulating factor 2 | 2.98 | 1.57 | 0.03 | 0.34 | 7.21 |
| IL12A | | interleukin 12A | 1.61 | 0.68 | 0.03 | 0.35 | 6.15 |
| BIRC5 | | baculoviral IAP repeat containing 5 | 2.97 | 1.57 | 0.05 | 0.40 | 6.32 |
| BRCA2 | | breast cancer 2, early onset | 5.41 | 2.44 | 0.01 | 0.26 | 5.35 |
| CDKN2C | | cyclin-dependent kinase inhibitor 2C | 2.36 | 1.24 | 0.01 | 0.26 | 6.92 |
| PCNA | | proliferating cell nuclear antigen | 1.71 | 0.77 | 0.01 | 0.26 | 9.32 |
| C1QA | | complement component 1, q subcomponent, A chain | 3.15 | 1.66 | 0.05 | 0.39 | 5.66 |
| CXCL10 | | chemokine (C-X-C motif) ligand 10 | 2.66 | 1.41 | 0.03 | 0.34 | 14.16 |
| ILF3 | | interleukin enhancer binding factor 3, 90kDa | 1.67 | 0.74 | 0.03 | 0.34 | 7.37 |
| C1S | | complement component 1, s subcomponent | 3.57 | 1.84 | 0.03 | 0.35 | 6.11 |
| LAG3 | | lymphocyte-activation gene 3 | 3.56 | 1.83 | 0.04 | 0.39 | 6.69 |
| GZMA | | granzyme A | 11.23 | 3.49 | 0.00 | 0.13 | 8.74 |
| BST1 | | bone marrow stromal cell antigen 1 | 2.05 | 1.04 | 0.05 | 0.39 | 5.44 |
| PRF1 | | perforin 1 | 2.34 | 1.23 | 0.03 | 0.34 | 7.25 |
| CD3G | | CD3g molecule, gamma | 1.58 | 0.66 | 0.01 | 0.23 | 9.76 |
| LOC490356 | | cytokine SCM-1 beta-like | 7.70 | 2.95 | 0.00 | 0.13 | 8.75 |
| LOC490629 | | transmembrane protease serine 9-like | 2.38 | 1.25 | 0.03 | 0.34 | 6.47 |
| GZMB | | granzyme B | 7.38 | 2.88 | 0.00 | 0.16 | 8.84 |
| TBX21 | | T-box 21 | 2.66 | 1.41 | 0.01 | 0.26 | 8.23 |
| LTA | | lymphotoxin alpha | 2.15 | 1.11 | 0.03 | 0.34 | 6.70 |
| TYMS | | thymidylate synthetase | 1.74 | 0.80 | 0.04 | 0.38 | 7.87 |
| KLRB1 | | killer cell lectin-like receptor subfamily B, member 1 | 3.49 | 1.80 | 0.00 | 0.16 | 7.55 |
| MKI67 | | antigen identified by monoclonal antibody Ki-67 | 5.30 | 2.40 | 0.01 | 0.23 | 6.40 |
| CDK1 | | cyclin-dependent kinase 1 | 3.49 | 1.80 | 0.04 | 0.39 | 6.07 |
| NKG7 | | natural killer cell group 7 sequence | 5.53 | 2.47 | 0.00 | 0.16 | 7.34 |
| IL13RA1 | | interleukin 13 receptor, alpha 1 | -1.67 | -0.74 | 0.00 | 0.15 | 8.16 |
| TNFRSF1A | | tumor necrosis factor receptor superfamily, member 1A | -1.53 | -0.61 | 0.04 | 0.39 | 8.72 |
| IL1RN | | interleukin 1 receptor antagonist | -1.95 | -0.96 | 0.05 | 0.39 | 9.86 |
| PDPN | | podoplanin | -4.54 | -2.18 | 0.03 | 0.34 | 6.74 |
| ADORA2A | | adenosine A2a receptor | -1.96 | -0.97 | 0.00 | 0.16 | 8.40 |
| IL1B | | interleukin 1, beta | -3.51 | -1.81 | 0.02 | 0.34 | 7.40 |
| CD36 | | CD36 molecule | -3.15 | -1.66 | 0.01 | 0.16 | 5.36 |
| PLAUR | | plasminogen activator, urokinase receptor | -2.49 | -1.31 | 0.00 | 0.16 | 8.56 |
| LRP1 | low density lipoprotein receptor-related protein 1 | | -2.44 | -1.29 | 0.00 | 0.16 | 7.54 |
| CSF2RB | colony stimulating factor 2 receptor, beta | | -2.35 | -1.23 | 0.04 | 0.38 | 7.54 |
| NCF4 | neutrophil cytosolic factor 4 | | -2.19 | -1.13 | 0.03 | 0.34 | 6.64 |
| TLR3 | toll-like receptor 3 | | -4.11 | -2.04 | 0.01 | 0.26 | 4.99 |
| SIGLEC1 | sialic acid binding Ig-like lectin 1 | | -2.66 | -1.41 | 0.02 | 0.34 | 6.73 |
| HCK | hemopoietic cell kinase | | -2.74 | -1.46 | 0.01 | 0.26 | 7.30 |
| SDC4 | syndecan 4 | | -2.23 | -1.16 | 0.02 | 0.34 | 10.96 |
| CMKLR1 | chemokine-like receptor 1 | | -2.68 | -1.42 | 0.03 | 0.34 | 6.51 |
| ITGA5 | integrin, alpha 5 | | -1.66 | -0.73 | 0.02 | 0.34 | 8.59 |
| LTBR | lymphotoxin beta receptor | | -2.09 | -1.06 | 0.01 | 0.22 | 6.08 |
| THBS1 | thrombospondin 1 | | -5.47 | -2.45 | 0.01 | 0.23 | 5.52 |
| IL1RAP | interleukin 1 receptor accessory protein | | -2.21 | -1.14 | 0.04 | 0.39 | 7.27 |
| CSF1R | colony stimulating factor 1 receptor | | -3.20 | -1.68 | 0.00 | 0.16 | 6.90 |
| ITGAM | integrin, alpha M | | -6.96 | -2.80 | 0.00 | 0.12 | 7.36 |
| CXCR3 | chemokine (C-X-C motif) receptor 3 | | -1.77 | -0.82 | 0.03 | 0.34 | 8.00 |
| TNFRSF9 | tumor necrosis factor receptor superfamily, member 9 | | 8.65 | 3.11 | 0.00 | 0.16 | 6.75 |
| CD55 | CD55 molecule, decay accelerating factor for complement | | -1.80 | -0.85 | 0.03 | 0.36 | 7.27 |
| SERPINB2 | serpin peptidase inhibitor, clade B (ovalbumin), member 2 | | -17.43 | -4.12 | 0.01 | 0.19 | 6.33 |
| AMICA1 | adhesion molecule, interacts with CXADR antigen 1 | | -1.63 | -0.70 | 0.05 | 0.40 | 8.63 |
| FADD | Fas (TNFRSF6)-associated via death domain | | -1.51 | -0.60 | 0.01 | 0.22 | 8.23 |
| CD9 | CD9 molecule | | -2.76 | -1.47 | 0.00 | 0.16 | 9.46 |
| FCRL2 | Fc receptor-like 2 | | -2.89 | -1.53 | 0.01 | 0.23 | 7.50 |
| IL6R | interleukin 6 receptor | | -1.98 | -0.99 | 0.01 | 0.16 | 6.12 |
| CEACAM1 | carcinoembryonic antigen-related cell adhesion molecule 1 | | -1.72 | -0.79 | 0.04 | 0.39 | 7.89 |
| PYCARD | PYD and CARD domain containing | | -2.27 | -1.18 | 0.04 | 0.39 | 6.36 |
| LOC102153988 | uncharacterized LOC102153988 | | -4.71 | -2.24 | 0.02 | 0.34 | 8.47 |
| LOC102154078 | uncharacterized LOC102154078 | | -7.20 | -2.85 | 0.00 | 0.16 | 7.13 |
| VSIR |  | | -1.89 | -0.92 | 0.00 | 0.12 | 9.24 |

p-Adj, p-Value adjustment; Ave. Log2 Exp., Average Log2 Expression
